# Intervention for school anxiety and absenteeism in children (ISAAC): Co-designing a brief parent-focused intervention for emotionally-based school avoidance

**DOI:** 10.1177/13591045231222648

**Published:** 2023-12-21

**Authors:** Brontë McDonald, Daniel Michelson, Kathryn J Lester

**Affiliations:** 1 1948 University of Sussex, Brighton, UK; 2 Department of Child and Adolescent Psychiatry, Institute of Psychiatry, Psychology and Neuroscience, King’s College London, UK; 3 NIHR Maudsley Biomedical Research Centre, South London and Maudsley NHS Foundation Trust and King’s College London, UK

**Keywords:** emotionally-based school avoidance, parent, intervention development, co-design

## Abstract

Emotionally-based school avoidance (EBSA) is an important driver of persistent school absenteeism and may have worsened in the context of COVID-19. This paper describes the development of a brief parent-focused psychosocial intervention with the goal to address the lack of accessible early interventions for EBSA. The developmental process used a person-based approach with two phases. In Phase 1, qualitative data were collected about intervention preferences and priorities from *N* = 10 parents and *N* = 7 practitioners in a series of co-design workshops. Phase 2 refined an intervention blueprint based on iterative consultations with *N* = 4 parents and *N* = 3 practitioners. Framework analysis was used to organise findings around key intervention parameters, including relevant mechanisms, content, and delivery methods needed to provide effective, acceptable and feasible support for families affected by EBSA. The resulting blueprint incorporates three online modules to be delivered over three weeks with each module consisting of psychoeducational videos, self-completed learning tasks and a corresponding coaching session. Respective module content includes: (i) self-care strategies to increase parent wellbeing and self-efficacy; (ii) parenting strategies to change behavioural patterns that maintain child distress and avoidance of school; and (iii) strategic communication strategies to increase the quality of home-school relationships. The blueprint has been developed into a full prototype for a forthcoming feasibility study.

## Plain language summary

Since the onset of the Covid-19 pandemic, there has been a noticeable increase in children missing school. One significant contributor to this rise in absences is Emotionally-Based School Avoidance (EBSA). EBSA refers to a situation where a child stays home from school due to feelings of anxiety and distress about attending. Accessing timely support for children with EBSA can be difficult due to lengthy waiting lists for child and adolescent mental health services. This paper outlines the creation of a new, brief psychosocial intervention aimed at helping parents support their child experiencing EBSA. The development process involved two phases. In the first phase, we collaborated with 10 parents and 7 practitioners to gather ideas about the kind of support families require, what should be included in the intervention, and how to make it practical for families to use. Based on these discussions, we formulated a plan for the new intervention. The second phase refined this plan of the intervention to ensure its suitability for families. The intervention is called ISAAC: Intervention for School Anxiety and Absenteeism in Children. ISAAC consists of three online modules to be completed by parents over a three-week period. Each module includes videos, reflection activities and homework tasks which help the parent learn a new skill or try a different way of responding to their child’s anxiety about attending school. Parents are also supported by a coach. The first module encourages parents to take care of their own wellbeing and manage stress. The second module helps parents to learn new ways to respond to their child's distress related to school attendance, while the third module provides guidance on effective communication with the child's school. The next step for this intervention is to see whether it is agreeable to parents and practical to deliver in the real-world.

## Introduction

Emotionally-Based School Avoidance (EBSA) refers to a constellation of emotional and behavioral difficulties in children that are specifically related to school attendance (Halligan & Cryer, 2022). EBSA adversely affects academic performance, family functioning and peer relationships (Kearney, 2008), and increases the likelihood of school dropout and long-term mental health problems (Fremont, 2003). The term is often used interchangeably with "school refusal," though EBSA is increasingly preferred by affected families and practitioners as it does not imply a deliberate choice to avoid school (Gregory & Purcell, 2014).

EBSA has been conceptualised within a broad ecological framework and linked to a range of individual, family and wider social risk factors (Melvin et al., 2019). The Covid-19 pandemic has amplified these risks by creating heightened Covid-related anxiety for children and parents, while disrupting learning and school routines (McDonald et al., 2022). Earlier studies suggest a population prevalence of around 1%–2% in school-aged children (Egger et al., 2003), with circumstantial evidence suggesting a more recent rise in EBSA cases as part of a general increase in persistent school absenteeism. Notably, data from the UK’s Department of Education (DfE) indicates that persistent absences in primary school (defined as missing >10% of school sessions) have increased from 11.2% in 2018 to 20.9% in 2022 (DfE, 2022).

EBSA typically first appears during the primary school years and prevalence peaks in secondary school (Havik et al., 2014). Early intervention is therefore important to mitigate more severe and persistent difficulties (Cook et al., 2017). However, effective psychosocial interventions for EBSA, such as those based on cognitive behavioural therapy (CBT) can be difficult to access in specialist mental health clinics (Elliot & Place, 2019) and service referral thresholds have been raised to manage demand since the pandemic (Huang & Ougrin, 2021). Thus, there is a need for resource-efficient interventions that can be accessed rapidly by families with primary-school aged children showing early signs of difficulty.

We conducted a prior study (McDonald et al., 2022) to understand better parents' and practitioners' preferences and priorities for EBSA interventions post-COVID. Participants emphasised the need for compassionate and non-judgmental support, particularly around changing patterns of “accommodating behaviours” that reduce children’s anxiety in the short-term but maintain distress and avoidance over time. These behaviours are a common focus of parenting work in CBT for child anxiety problems (e.g., Lebowitz et al., 2014). However, many parents in our study felt that practitioners insufficiently acknowledged the difficulty of changing established behavioural routines. This underscores the importance of validating parents’ experiences; addressing parents’ own mental well-being; and addressing strained relationships that may develop when parents and school staff differ in their expectations of what needs to change (and how).

The present study drew directly on these formative findings and the wider evidence base. Working collaboratively with parents and practitioners, we aimed to co-design a scalable, community-based intervention with the overall goal of improving school attendance and reducing associated distress for primary school-aged children affected by EBSA. The intervention was intended to increase parents’ competencies in three key areas implicated in EBSA: (i) using self-care to increase parent wellbeing and self-efficacy; (ii) modifying accommodating behaviour patterns that maintain child’s avoidance of school; and (iii) communicating more constructively to reduce conflict and increase collaboration with school staff (McDonald et al., 2022). The specific research questions were:

- What are the most relevant intervention targets and what content, and materials are needed to address these?
- How should the intervention content be delivered to minimise burden and enhance intervention coherence and self-efficacy?
- Who should deliver the intervention to ensure it is credible and engaging?
- When and how often should the intervention be delivered to ensure it is efficient, feasible yet effective?

## Methods

### Study design

We employed an iterative person-based approach to co-design the intervention (Yardley et al., 2015). Phase 1 consisted of workshops with parents and practitioners to inform an initial intervention blueprint built around standardised intervention descriptors from the Template for Intervention Description and Replication framework (TIDieR, Hoffman et al., 2014). In Phase 2, the provisional blueprint was refined through individual consultations with a subset of Phase 1 participants. Ethical approval was obtained from a Research Ethics Committee at the University of Sussex (Reference: ER/BM333/8).

### Participants

Eligible parents were living with an index child who (i) was currently enrolled in a state-funded mainstream primary school in Sussex and (ii) had experienced EBSA in the previous academic year (2020-21), based on the parent’s own report (i.e., we did not formally assess EBSA or seek corroborating evidence from schools but instead asked parents to nominate if their child had experienced difficulties and anxieties attending school in 2019–2022). The final sample comprised 10 parents, all of whom were biological parents (nine mothers, one father) and each child attended a different school in Sussex. Three of these parents had participated in a previous qualitative study by the same authors (McDonald et al., 2022) and had provided consent for further contact. Seven additional parents were recruited through targeted advertisements via social media. See Table 1 for further details.

**Table 1.** Descriptive Characteristics of Parent Participants.

	Phase one		Phase two	
	*N* (%)	Mean (SD)	*N* (%)	Mean (SD)
Parent				
Gender				
Female	9 (90%)		3 (75%)	
Male	1 (19%)		1 (25%)	
Ethnicity				
White British	10 (100%)		4 (100%)	
Household income				
<£29,999	3 (30%)		—	
£30,000-£49,999	2 (20%)		1 (25%)	
>£50,000	4 (40%)		3 (75%)	
Not disclosed	1 (10%)		-	
Child				
Gender				
Female	8 (80%)			
Male	2 (20%)			
Eligible for free school meals				
Yes	2 (20%)		1 (25%)	
No	8 (80%)		3 (75%)	
Receiving additional support^a^				
Yes – anxiety	2 (20%)		—	
Yes – Autism, PDA, ADHD	1 (10%)		1 (25%)	
No	7 (70%)		3 (75%)	
Persistently absent^b^				
Yes (missed >10% of total sessions)	4 (40%)		2 (50%)	
No (missed < 10% of total sessions)	6 (60%)		2 (50%)	
Age	—	9.10 (1.75)	—	10.33 (0.58)

^a^Additional support provided for learning needs without an Education Health Support Plan.

^b^Met the DfE (2022) criteria for persistent absences in spring term 2022, i.e., parent reported that child was absent for more than 10% of all school sessions during the term.

Eligible practitioners were education and mental health practitioners, based in Sussex with professional experience of working with families affected by EBSA. We first approached the twelve practitioners who participated in our earlier formative study (McDonald et al., 2022), out of which four practitioners agreed to take part in the current study. In addition, three new practitioners joined the study after being recruited through additional contacts with local authorities and educational psychology services. We focused the latter recruitment process on local authorities in Sussex that exhibited the highest persistent absence rates (DfE, 2022).

The combined sample of practitioners comprised three headteachers, one deputy headteacher, one educational psychologist, one education mental health practitioner, and one education, behaviour and attendance support officer.

### Procedure

#### Phase 1

Prospective participants accessed a participant information sheet and consent form via a weblink sent by email or through digital recruitment materials. Workshop dates were determined based on participants' availability. Compensation of £15/hour was provided to participants for attending a workshop, along with reimbursement for travel expenses and/or childcare costs when attending in person.

Three co-design workshops were held during April and May 2022: one in-person parents’ workshop (*n* = 6), one online parents’ workshop (*n* = 4), and one online practitioners’ workshop (*n* = 7). A priori decision was made to focus on parent-directed interventions based on recent research demonstrating their cost-effective nature in reducing child anxiety and related behaviours (Creswell et al., 2021). The starting point for developing the parent-directed intervention was an earlier study that identified three modifiable risk factors (parental stress and wellbeing; parental accommodation of child anxiety; and home-school communication) as key intervention targets with the potential to reduce EBSA. Workshop participants reviewed the relevance of these targets and discussed suitable practice elements. Potential intervention formats were then explored, drawing from examples of brief, low-intensity psychosocial interventions (Gonsalves et al., 2021; Sung et al., 2021).

Interactive activities were used to facilitate discussion around each research question. Activities included brainstorming, completing a “pros and cons” table, and voting on potential intervention features. In-person workshops took place in a university seminar room using physical tools such as post-it notes and flipcharts. Online workshops were conducted via Zoom, utilising a shared Jamboard. Participants' contributions were audio-recorded, and physical artefacts (e.g., Post-It notes, flipchart paper) were collected by a researcher.

Practitioners received a summary of the parents' priorities and ideas for each research question prior to their online workshop. The workshop proceeded in a similar interactive format as used with parents.

#### Phase 2

A series of individual consultations (each lasting 30–60 mins) were carried out with a subset of Phase 1 participants in October-November 2022 (*n* = 4 parents, *n* = 1 educational psychologist, *n* = 1 headteacher and *n* = 1 deputy headteacher). Contributors received a £15 e-voucher as compensation for their time. Participants shared their thoughts on the acceptability of the intervention components, the clarity and usefulness of draft materials (including draft written materials and activities for each module); the delivery approach, potential for engagement by parents, and suggested improvements. The intervention blueprint and materials were refined based on these consultations.

### Analysis

Framework analysis (Gale et al., 2013) was conducted sequentially on the qualitative data from workshops and consultations. A deductive approach was taken to develop the initial framework with the research questions and TIDieR descriptors creating framework categories. The analysis followed the steps of familiarisation, coding, charting themes, mapping and interpreting the data, with analysis conducted after each workshop/consultation to inform subsequent data collection activities (Gale et al., 2013). The analysis was led by BM, a PhD researcher with prior experience as a primary school teacher, who employed a critical realist standpoint and incorporated personal insights from their teaching background. Regular discussions were held with the senior co-authors (PhD co-supervisors) to scrutinise assumptions and interpretations.

## Results

### What are the most relevant intervention targets and what content, and materials are needed to address these?

The focal areas established in earlier formative work were first presented to stakeholders: (i) enhancing self-care strategies to increase parent wellbeing and self-efficacy; (ii) modifying accommodating behavioural patterns that can maintain child distress and avoidance of school; and (iii) teaching strategic communication strategies to improve quality of home-school relationships.

#### Module one: Self-care strategies to increase parent wellbeing and self-efficacy

Parents recognised the importance of taking care of themselves in order to better respond to their child’s anxiety. Practitioners recommended the intervention start with this module, so that parents felt prepared to then address their child’s anxiety. Practitioners also suggested specific techniques like breathing exercises, school routine planning, and facilitating communication with other parents in similar situations that they had found to be effective in enhancing parent wellbeing (Da Paz and Wallander et al., 2017).

“I think for parents to be supported as a first step in this process is great, because until parents feel supported and confident, I think the next bit is a very big ask.” Parent consultation

Thus, module one explains the importance of parental self-care and provides guidance on recognising signs of stress and use of breathing exercises, muscle relaxation, and visualisation techniques. Additionally, the module covers long-term self-care strategies, such as positive self-talk, engaging in activities outside of family life, maintaining physical wellbeing, planning ahead for stressful moments, and nurturing connections with others.

#### Module two: Parenting strategies to change accommodating behavioural patterns that maintain child distress and avoidance of school

Parents wanted more guidance on strategies to use when their child was distressed about attending school. Practitioners recommended including psychoeducation on anxiety and the avoidance cycle as the initial step to teach that children’s confidence to cope with distress can be undermined by parent accommodations (e.g. having the child stay home after exhibiting distress about attending school). Consequently, we integrated psychoeducational content on anxiety and the avoidance cycle, referencing existing sources (Cartwright-Hatton et al., 2011; Lebowitz et al., 2014). The module then focused on helping parents to reduce accommodating behaviours and replace them with supportive strategies. Module content covered validating their child’s feelings, encouraging them to develop a plan to manage their child’s anxiety without accommodating behaviors, modelling healthy coping strategies, and advice on seeking professional assistance should their child’s anxiety worsen.

“Looking back, one thing that we did that was an error, we accommodated really heavily because we thought if she was comfortable she would chill out and then be able to do things again whereas in fact the route that took us down was she knocked off one thing after another after one and it just cascaded and made everything harder.” Parent consultation

Practitioners emphasised the importance of compassionately offering adaptive parenting strategies proven to reduce child anxiety, as opposed to blaming and criticising parents.

#### Module three: Strategic communication strategies to increase quality of home-school relationships

Parents acknowledged the significance of positive home-school relationships and wanted content on effective communication strategies to allow them to feel more at ease and confident communicating with their child’s school. Practitioners suggested adopting a solution-focused approach to school communications and saw this as one way of enhancing the home-school relationship.

Content for module three therefore incorporated practitioners' suggestions and relevant research (e.g., Tobias, 2019) with respect to practical solution-focused communication. This included preparation for meetings/communications, e.g., by identifying anxiety triggers, clear goals and desired outcomes; encouraging a collaborative approach to addressing problems and agreeing a shared action plan; monitoring progress against this action plan, remaining open to adjustments, and maintaining open lines of communication.

“I always ask parents to make notes before meeting so they can ensure all their points are discussed as these meetings can get very emotional.” Practitioner workshop

### How should the intervention content be delivered to minimise burden and enhance intervention coherence and self-efficacy?

#### Content delivery through videos

Parents described the drawbacks of previous interventions, such as reading lengthy text or listening for extended periods and expressed a preference for slides or animations accompanied by an audio soundtrack.

“I struggle listening to one person for a long time... It would be good to have a level of interactivity, having something to click on to make it happen.” Parent consultation

Accordingly, we structured the intervention around four short videos per module in line with other interventions showing that such formats can have positive effects on user engagement, usability, and enjoyment (Dunn et al., 2022). The first provided a rationale for the weekly topic. The second offered psychoeducation on how the topic relates to their child’s EBSA. The remaining videos focused on practical ways to incorporate the content into family life.

Practitioners recommended incorporating vignettes featuring parents and children with EBSA, as they enhance knowledge retention, and motivation (Ritterband et al., 2009). We developed the vignette “Alex and Sam'” which depicted the challenges of EBSA and incorporated worked examples of the suggested strategies within each module (Appendix 1). It was consistently integrated across all modules to connect psychoeducation with real-life scenarios.

#### Reflection tasks

Practitioners recommended incorporating interactive activities to enhance engagement and understanding. Consequently, the intervention includes "reflection tasks" that parents complete after each video. For instance, in module one, parents are asked to recall a recent stressful experience and check off related symptoms. They are then prompted to reflect on their current strategies for managing these feelings.

“Structured reflection tasks would be good for parents to complete to help them understand and engage with the learning.” Practitioner workshop

#### Home learning tasks

Parents discussed the requirement for home-based practice to be “*straightforward and not too time-consuming.*” Moreover, parents disliked the term “homework” We decided to conclude each module by asking parents to create a plan to complete a small “learning task” that applied the module’s content.

“I like the idea of homework, I don’t know if you’d call it homework.... but like if in a session you were taught about different methods to manage your stress and try them out that week.” Parent workshop

For example, module one instructs parents to identify a “pressure point” when feeling stressed and to try using one of the stress management techniques outlined in the module. These tasks were confirmed to be manageable by parents during consultations. Parents emphasised the need for accountability in completing the tasks. Fabiano et al. (2009) found that the inclusion of coaches in a parent training programme resulted in higher homework completion rates. Practitioners suggested that *“planning these activities with someone would be helpful*.” Therefore, coaching sessions after each module aimed to support the completion of the learning task.

#### Blended delivery

Stakeholders discussed the pros and cons of different modes of delivery, including in-person group meetings, online meetings, and self-guided versus “blended” approaches (i.e., combining interpersonal support with an online platform; Sanderson et al., 2020). While parents identified benefits of connecting with other parents in group therapy, they also noted that practical barriers like child-care and work could limit regular attendance. A brief, blended programme with a mixture of online content and remote guidance was seen to hold practical advantages.

“There is only one sort of person who can access it [in-person meetings] and it [would typically be] mums and mums that don’t work.” Parent consultation

As such, the planned intervention is intended to be delivered primarily online and accessed through a webpage. Parents’ desire for connection with other parents is addressed indirectly, by including suggestions for parents to connect with others in school settings and through online support groups.

#### Coaching

Coaching is often included in online interventions due to its positive impact on participant engagement and outcomes (Kenwright et al., 2004). The consultations therefore explored how coaching could be used within the intervention.

Parents and practitioners recommended a brief discussion with a coach after each module to ensure completion, to support understanding and to address any technical issues. Parents also wanted a coach to be able to signpost them to additional relevant information and sources of support. Preferences for coaching mode (text, phone, or Zoom) varied among parents.

“You know it might be helpful to be signposted to another organization course kind of thing because there's so many of them, I just can’t keep track of all of them because it’s all different” Parent workshop

For this intervention, the coach will conduct an "onboarding call" similar to Gonsalves et al. (2021) to introduce the modules and provide a tutorial on accessing the intervention materials. After completing each module, participants will receive a 20–30-min coaching session via their preferred format to review module content, address questions and review the learning task.

### Who should deliver the intervention to ensure it is credible and engaging?

Favoured attributes of the coach included social skills, kindness, active listening, empathy, non-judgmental attitude, and a calm demeanor alongside strong background knowledge in mental health and anxiety specifically. Parents preferred the coach to be someone who was unconnected to their child’s school, as they felt this would allow for more open discussions on sensitive topics.

### When and how often should the intervention be delivered to ensure it is efficient, feasible yet effective?

Parents wanted to avoid an excessively time-consuming intervention and agreed that dedicating *“an hour a week is certainly doable.”* It was decided that each topic would be covered in an approximately 30–60 minute-long module, to be completed weekly. Parents emphasised the need for flexibility due to the unpredictability of family life. Consequently, we allowed parents to reschedule coaching appointments and extend the time between each module if necessary.

## Discussion

This study employed stakeholder workshops and consultations with the aim to develop a scalable, community-based intervention that improves school attendance and reduces associated distress for primary school-aged children affected by EBSA. We found strong support for three focal content areas, covering parental stress and wellbeing, parental accommodation of child anxiety, and home-school communication. The intervention blueprint incorporates three corresponding modules comprised of psychoeducational videos, self-directed reflection tasks, and additional home learning activities. Each module is delivered online in self-guided format and followed by a coaching session to discuss module content and support generalisation into real-life scenarios. We have named the programme “Intervention for School Anxiety and Absenteeism in Children” (ISAAC) and summarised its key features in Table 2.

**Table 2.** Intervention for School Anxiety and Absenteeism in Children (ISAAC) Blueprint.

	
WHY	
Describe any rationale, theory, or goal of the elements essential to the intervention	*Eligibility criteria:* Parent(s) of 1) child (ren) enrolled in a mainstream primary school experiencing emotional distress associated with school attendance 2) has access to an internet-enabled device and competency in English, as required to engage with the content of the intervention *Goal:* To reduce EBSA through short-term outcomes of reducing parent stress, lowering children’s anxiety around attending school, reducing parent accommodations and improving home-school communication *These outcomes will be achieved through:* Self-care strategies to increase parent wellbeing and self-efficacy; parenting strategies to change accommodating behavioural patterns that maintain child distress and avoidance of school; and solution-focused strategies to improve home-school communication
WHAT	
Materials: Describe any physical or informational materials used in the intervention, including those provided to participants or used in intervention delivery	An online webpage with links to three modules corresponding to each topic area. Each module comprises of an interactive worksheet with four videos, reflection tasks and one home learning task
Procedures: Describe each of the procedures, activities, and/or processes used in the intervention	The videos aim to provide psychoeducational content on three topics known for their effectiveness in previous interventions In the first module, which focuses on managing parents’ stress and wellbeing, the videos explain the significance of maintaining wellbeing, especially when caring for an anxious child (Palmer et al., 2023). They will also guide parents on recognising stress indicators and introduce specific evidence-based techniques, such as breathing exercises and muscle relaxation to alleviate stress and promote overall wellbeing (Da Paz & Wallander, 2017) In the second module, centred on the parents managing their child’s anxiety, the videos address how a child’s anxiety about school can contribute to an avoidance cycle (Cartwright-Hatton et al., 2011). They will explore accommodating behaviors and their role in perpetuating this cycle, as well as provide alternative strategies for parents, including confidence-building statements (Lebowitz et al., 2014) In the third module, which pertains to parents communicating effectively with their child’s school, the videos emphasise the importance of establishing a positive relationship with the school to support their child’s attendance (Smith et al., 2020). They will introduce solution-focused communication as a means to enhance this relationship and offer various solution-focused strategies that parents can utilise before, during, and after meetings to bolster their confidence when engaging with the school (Tobias, 2019) The reflection tasks support and extend psychoeducation content and provide opportunities to apply content to the parent’s own experiences. The home learning task will encourage parents to put into practice the guidance and strategies learned. Parents will be provided with a coach. The coach will introduce the intervention via an ‘onboarding’ session and will meet with the parent after each module to review module content, learning task progress and signpost to additional resources
WHO PROVIDED	
For each category of intervention provider describe their expertise, background and any specific training given	The coach will be unconnected to the child’s school, have relevant mental health expertise, be trained in active listening and be kind, non-judgemental and empathetic
HOW	
Describe the modes of delivery of the intervention and whether it was provided individually or in a group	Three self-guided online modules hosted on a webpage. Parents will interact with a coach via zoom, phone and/or text
WHERE	
Describe the type(s) of location(s) where the intervention occurred, including any necessary infrastructure or relevant features	Module content delivered online. Coaching via Zoom, text or phone
WHEN and HOW MUCH	
Describe the number of times the intervention was delivered and over what period of time including the number of sessions, their schedule, and their duration, intensity or dose	One module per week plus coaching session but with some flexibility to extend time taken to complete module and reschedule coaching session. Each module to last 30–60 minutes. Coaching sessions to last 20–30 minutes

Many children with mental health difficulties and their families are unable to access the mental health support services they need (Huang & Ougrin, 2021). Kazdin (2019) argues that this need-to-access gap is unlikely to change until there is a significant shift away from interventions requiring a face-to-face format and delivery by an “expert” with extensive training. Considering this, the ISAAC programme has been designed as a brief, blended intervention for EBSA. The three self-guided modules are considered to be the primary mechanism of change. Each module is designed to help the parent learn a new skill (e.g. relaxation, solution-focused communication), and/or try a different way of responding to their child’s distress (e.g. by changing accommodating behaviours) through psychoeducation videos, reflection tasks and specific homework suggestions. This mixed content format is well-established in parenting interventions for child mental health difficulties (e.g. Dunn et al., 2022) and has been shown to enhance parent engagement (Van Stappen et al., 2019). After completing the module and homework, the parent meets with a coach. Support from a coach is designed to enhance the efficacy of the intervention by helping the parent practice the skills they have learned in their daily lives, reflect on the impact of doing so, and troubleshoot any barriers to home practice and generalisation.

Interventions delivered remotely are a useful tool for treating emerging mental health problems in children and there is good evidence that they can be as effective as interventions delivered in-person (Rooksby et al., 2015). For example, internet-delivered CBT (iCBT) programmes for child anxiety have performed equivalently or favourably compared to waitlist control conditions and traditional in-person CBT (Rooksby et al., 2015), and are acceptable to parents (March et al., 2018). However, program adherence and drop-out are often reported challenges in implementing iCBT programmes (Christensen et al., 2009). Recent research suggests that the presence of synchronous support significantly moderates treatment outcomes for iCBT (Venturo-Conerly et al., 2022) and that program adherence is improved when an intervention includes even a brief amount of clinician support (Berg et al., 2020). For this reason, ISAAC has been designed to include support from a coach at the outset of the programme and following the completion of each module. However, remaining mindful of both the preferences of our participants and the necessity of moving away from intervention models that rely on access to “experts”, we argue that the coach need not be a highly qualified therapist (Gonsalves et al., 2021; Kazdin, 2019). Parents in this study prioritised empathy, social skills and a non-judgemental attitude over mental health qualifications in a coach, aligning with Baumeister et al.'s (2014) findings emphasising the coach’s efficacy over qualifications. Thus, we recommend that coaches receive training in active listening, effective coaching techniques, and possess foundational knowledge in child anxiety.

Previous research also provides confidence that brief interventions can help reduce mental health difficulties in children (Malik et al., 2022). A recent meta-analysis (Schleider & Weisz, 2017) and review (Schleider et al., 2019) demonstrated that even single session interventions (SSIs) produce significant benefits for youth mental health difficulties. Significant treatment effects emerged irrespective of severity of disorder, diagnostic status, degree of therapist support and delivery format (e.g. web-based vs. face-to-face). While treatment effects for SSIs were slightly smaller compared to traditional, multi-session psychosocial therapy, brief interventions comprising single or very limited numbers of sessions, have significant advantages in terms of potential for scalability, accessibility and larger-scale impact (Schleider et al., 2019). With this in mind, we anticipate that the online modules within ISAAC should take parents no more than 3 hours to complete and web-based delivery means that the programme can be made openly available for parents to complete at a time and location of their choosing.

### Strengths and limitations

ISAAC has been developed against a backdrop of increased persistent school absenteeism and a probable rise in cases of EBSA in the aftermath of the pandemic (McDonald et al., 2022). ISAAC offers a resource-efficient intervention that can be scaled quickly and which circumvents current bottlenecks in accessing psychosocial interventions through specialist mental health clinics allowing parents to access support rapidly.

The use of extensive co-design in the development of ISAAC is also a significant strength. Past research indicates that including stakeholders in the process of intervention development improves relevance, implementation and outcomes (Craig et al., 2018). ISAAC is not only grounded in the preferences and priorities of its intended users (parents) but also incorporates the expertise of practitioners to enhance acceptability and effectiveness (O’Cathain et al., 2017).

Our study included an equal division of parents on either side of the “persistently absent” threshold (i.e., missing more than 10% of school sessions), which is set by the UK Department for Education. In the early stages of EBSA, parents may succeed in getting their child to attend school despite significant behavioural and emotional difficulties related to the child’s anticipated and/or actual attendance. As these difficulties intensify, the frequency of school absences typically begins to rise. By including parents with index children who were distressed by school but not yet persistently absent, we were able to gain insights across a wide spectrum of experience.

This study has some notable limitations. First, even though study participation was open to parents of all genders, it was mainly mothers who participated. Research has found that mothers tend to be more actively engaged in their children’s schooling (Lewis & Lamb, 2003), which increases the likelihood that they will take part in school-based research (Räty et al., 2009). Mothers may also face particular challenges related to managing EBSA, on account of being more likely take children to school compared to fathers (Barker, 2011). Second, our sample consisted entirely of white British participants, which limits the generalisability to more ethnically diverse populations. Given the pandemic context in which ISAAC was developed, it is notable that several studies have reported relatively greater psychosocial impacts of COVID-19 for families from racially minoritised groups (Batra et al., 2022; McIvor et al., 2022; Whitaker et al., 2021), which may in turn affect risks for the onset and persistence of EBSA. Future research should endeavour to capture the perspectives of fathers and other underrepresented groups in intervention testing and refinement.

Third, it is possible that the practitioners who took part in the current study may be unrepresentative of wider professional views. That said, we specifically recruited practitioners with professional experience of EBSA and we identified broadly similar views between practitioners who were recruited specifically for the current study, and those who reprised their involvement from an earlier linked study.

### Conclusions

The ISAAC programme offers a brief, blended intervention for parents with the aim to improve school attendance and reduce associated distress in primary school-aged children with EBSA. The next step will be to ascertain the feasibility and acceptability of delivering ISAAC in the real-world and to explore it’s potential to deliver on reducing distress and improving school attendance in children with EBSA.

## Data Availability

The detailed coding framework used in this study is available on request from the corresponding author. Participants did not provide permission to share raw data (i.e., unprocessed transcripts) outside of the core research team.

## Appendix

### Appendix 1: Example Extract of Alex and Sam Vignette

Let’s start by looking at Alex’s situation with their child. Alex’s 7-year-old son, Sam has been anxious about going to school. Sam has been worrying about getting questions wrong at school and getting told off by his teacher. He is particularly worried about the spelling test on Mondays. This makes him feel like he has a tummy ache, and he often feels afraid.

Sam understandably doesn’t want to feel this way and wants to avoid this feeling. One of the ways he tries to stop this feeling is by avoiding the anxiety-provoking situation altogether; school. So to avoid school Sam acts out and gets upset when asked to put on his school uniform until eventually Alex helps get him dressed. Sam then often refuses to get into the car to go to school. Thinking about going to school makes him feel tense. His distress about school can escalate to crying and clinging to Alex at the school gates until Alex walks with him into school. Sam gets so distressed that sometimes Alex keeps him off school that day to help ease this anxiety and help him feel calm again.

However, avoiding school only provides temporary relief for Sam. The next day he is back to square one. This unfortunately creates, what is often called the school avoidance cycle, as staying at home only provides temporary relief from the anxiety and over time it can lead to an increase in anxiety and school avoidance behaviours.

Did you spot some of the ways in which Sam’s anxiety impacted on his body, thoughts, feelings and behaviour? Anxiety can look very different for different children but it is possible that your child’s anxiety may show itself in similar ways.
